# Supplementary material for: From sequence to enzyme mechanism using multi-label machine learning
Source: BMC Bioinformatics. 2014 May 19;15:150. doi: 10.1186/1471-2105-15-150 (PMC4229970; doi:10.1186/1471-2105-15-150)
Supplement: Additional file 2 — Java code of ml2db. Additional file ml2db_code.tar.gz contains the Java source code to run the multi-label machine learning experiments and save the results to database. The code’s Javadoc is included. [file 1471-2105-15-150-S2.zip › additional file 2/ml2db/ml2db/doc/main/ml2db/mulan/learn/crosseval/CrossExperimenter.html]

CrossExperimenter


JavaScript is disabled on your browser.


- Overview
- Package
- Class
- Use
- Tree
- Deprecated
- Index
- Help

- Prev Class
- Next Class

- Frames
- No Frames

- All Classes

- Summary:
- Nested |
- Field |
- Constr |
- Method

- Detail:
- Field |
- Constr |
- Method


main.ml2db.mulan.learn.crosseval

## Class CrossExperimenter

- java.lang.Object
- - main.ml2db.mulan.learn.crosseval.CrossExperimenter

- Direct Known Subclasses:
  :   TrainTestExperimenter

  ---

    

  ```
  public class CrossExperimenter
  extends java.lang.Object
  ```

  Using Mulan (multi-label machine learning), this class initialises and
  executes either Train-Test evaluation (if two different arff records are
  given) (calling `MulanTrainTestEvaluator`) or cross-evaluations (calling
  `MulanCrossEvaluator`) if only one arff record is given. Initialisation
  includes verifying input (arff and xml files) and checking output locations
  (for saving results to file and database).

  Version:
  :   14 Jun 2010

  Author:
  :   Luna De Ferrari ldf(at)st-andrews.ac.uk

- - ### Constructor Summary

    Constructors

    Constructor and Description

    `CrossExperimenter(DbManagers dbMan,
    int trainArffRecordId,
    MulanLearner learner,
    java.lang.String resultsFolder)`

    `CrossExperimenter(IdMultiLabelInstances trainSet,
    MulanLearner learner,
    DbManagers dbMan,
    java.lang.String resultsFolder)`
  - ### Method Summary

    Methods

    | Modifier and Type | Method and Description |
    | --- | --- |
    | `java.util.Vector<java.lang.Object[]>` | `evaluateAndSave(int folds, boolean savePredictions, boolean saveTrueNegatives, boolean stratify)` Cross validates returns evaluation results. |
    | `CrossInputManager` | `getInputManager()` |
    | `MulanLearner` | `getLearner()` |
    | `CrossOutputManager` | `getOutputManager()` |
    | `int` | `getTestArffId()` |
    | `int` | `getTrainArffId()` |
    | `static void` | `main(java.lang.String[] args)` |
    | `static void` | `run(int[] arffFilesIds, java.lang.String resultsDbPropsPath, int folds, java.util.Vector<MulanLearner> learners, boolean savePredictions, boolean saveTrueNegatives, boolean stratify, int numberOfCrossEval)` Run a cross-evaluation of several ARFF files (using one or several algorithms, as needed) |
    | `void` | `setInputManager(IdMultiLabelInstances trainSet)` |
    | `void` | `setInputManager(int trainArffRecordId, DbManagers dbManagers)` |
    | `void` | `setOutputManager(uk.ac.standrews.utils.main.database.DbManager resultsDbManager, java.lang.String resultsFolder, uk.ac.standrews.utils.main.database.Table table)` |
    | `void` | `setTestArffId(int testArffId)` |

    - ### Methods inherited from class java.lang.Object

      `equals, getClass, hashCode, notify, notifyAll, toString, wait, wait, wait`

- - ### Constructor Detail


    - #### CrossExperimenter

      ```
      public CrossExperimenter(DbManagers dbMan,
                       int trainArffRecordId,
                       MulanLearner learner,
                       java.lang.String resultsFolder)
      ```


    - #### CrossExperimenter

      ```
      public CrossExperimenter(IdMultiLabelInstances trainSet,
                       MulanLearner learner,
                       DbManagers dbMan,
                       java.lang.String resultsFolder)
      ```
  - ### Method Detail


    - #### evaluateAndSave

      ```
      public java.util.Vector<java.lang.Object[]> evaluateAndSave(int folds,
                                                         boolean savePredictions,
                                                         boolean saveTrueNegatives,
                                                         boolean stratify)
      ```

      Cross validates returns evaluation results.


    - #### getInputManager

      ```
      public CrossInputManager getInputManager()
      ```


    - #### getLearner

      ```
      public MulanLearner getLearner()
      ```


    - #### getOutputManager

      ```
      public CrossOutputManager getOutputManager()
      ```


    - #### getTestArffId

      ```
      public int getTestArffId()
      ```

      Returns:


    - #### getTrainArffId

      ```
      public int getTrainArffId()
      ```

      Returns:


    - #### setInputManager

      ```
      public void setInputManager(IdMultiLabelInstances trainSet)
      ```


    - #### setInputManager

      ```
      public void setInputManager(int trainArffRecordId,
                         DbManagers dbManagers)
      ```


    - #### setOutputManager

      ```
      public void setOutputManager(uk.ac.standrews.utils.main.database.DbManager resultsDbManager,
                          java.lang.String resultsFolder,
                          uk.ac.standrews.utils.main.database.Table table)
      ```


    - #### setTestArffId

      ```
      public void setTestArffId(int testArffId)
      ```


    - #### main

      ```
      public static void main(java.lang.String[] args)
      ```


    - #### run

      ```
      public static void run(int[] arffFilesIds,
             java.lang.String resultsDbPropsPath,
             int folds,
             java.util.Vector<MulanLearner> learners,
             boolean savePredictions,
             boolean saveTrueNegatives,
             boolean stratify,
             int numberOfCrossEval)
      ```

      Run a cross-evaluation of several ARFF files (using one or several
      algorithms, as needed)

      Parameters:
      :   `arffFilesIds` - a list of ARFF file identifiers in the "arff" database table
      :   `learners` -


- Overview
- Package
- Class
- Use
- Tree
- Deprecated
- Index
- Help

- Prev Class
- Next Class

- Frames
- No Frames

- All Classes

- Summary:
- Nested |
- Field |
- Constr |
- Method

- Detail:
- Field |
- Constr |
- Method
